# Immune Response and Immune Checkpoint Molecules in Patients with Rectal Cancer Undergoing Neoadjuvant Chemoradiotherapy: A Review

**DOI:** 10.3390/cimb45050285

**Published:** 2023-05-22

**Authors:** Ioannis M. Koukourakis, Kalliopi Platoni, Dina Tiniakos, Vassilis Kouloulias, Anna Zygogianni

**Affiliations:** 1 Radiation Oncology Unit, 1st Department of Radiology, School of Medicine, Aretaieion University Hospital, National and Kapodistrian University of Athens (NKUOA), 11528 Athens, Greece; koukourioannis@gmail.com (I.M.K.); azygogianni@med.uoa.gr (A.Z.); 2 Medical Physics Unit, 2nd Department of Radiology, School of Medicine, Attikon University Hospital, National and Kapodistrian University of Athens, 12462 Athens, Greece; 3 Department of Pathology, School of Medicine, Aretaieion University Hospital, National and Kapodistrian University of Athens, 11528 Athens, Greece; dtiniak@gmail.com; 4 Translational and Clinical Research Institute, Faculty of Medical Sciences, Newcastle University, Newcastle upon Tyne NE2 4HH, UK; 5 Radiotherapy Unit, 2nd Department of Radiology, School of Medicine, National and Kapodistrian University of Athens, 12462 Athens, Greece; vkouloul@med.uoa.gr

**Keywords:** rectal cancer, radiotherapy, immune checkpoint, HLA, aminoacid metabolism, prognosis

## Abstract

It is well-established that tumor antigens and molecules expressed and secreted by cancer cells trigger innate and adaptive immune responses. These two types of anti-tumor immunity lead to the infiltration of the tumor’s microenvironment by immune cells with either regulatory or cytotoxic properties. Whether this response is associated with tumor eradication after radiotherapy and chemotherapy or regrowth has been a matter of extensive research through the years, mainly focusing on tumor-infiltrating lymphocytes and monocytes and their subtypes, and the expression of immune checkpoint and other immune-related molecules by both immune and cancer cells in the tumor microenvironment. A literature search has been conducted on studies dealing with the immune response in patients with rectal cancer treated with neoadjuvant radiotherapy or chemoradiotherapy, assessing its impact on locoregional control and survival and underlying the potential role of immunotherapy in the treatment of this cancer subtype. Here, we provide an overview of the interactions between local/systemic anti-tumor immunity, cancer-related immune checkpoint, and other immunological pathways and radiotherapy, and how these affect the prognosis of rectal cancer patients. Chemoradiotherapy induces critical immunological changes in the tumor microenvironment and cancer cells that can be exploited for therapeutic interventions in rectal cancer.

## 1. Introduction

The role of white blood cells and major histocompatibility antigens in the rejection of tumors implanted in experimental animals has been well-established for several decades [1]. Both the innate, mediated by monocytes and NK-cells, and the adaptive immune response, mediated by B- and T-lymphocytes, are involved in the immune system surveillance over tumors. Τumor antigens activate dendritic cells that educate T-cells to elicit antigen-specific cytotoxic responses. Cytokines and chemokines, secreted by cancer cells, trigger innate and adaptive immune responses, also acting as chemotactic stimuli to attract inflammatory cells in the tumor environment [2]. 

The nature of the response that follows depends, however, on many cancer-cell- and microenvironment-dependent parameters. Differentiation and phenotypic changes towards specific monocyte and T-cell subpopulations with contrasting activities, promoting immune tolerance or cytotoxic anti-tumor effects, will eventually define the prevalence of a regulatory or cytotoxic immune response. This complex cellular and molecular interplay has been a matter of extensive research through the years, mainly focusing on tumor-infiltrating lymphocytes (TILs) and monocytes and their subtypes, the expression of immune checkpoints and other immune-related molecules by both immune and cancer cells, and how this affects the prognosis of patients and the outcome of chemotherapy and radiotherapy (RT) [3,4].

During the last two decades, immunotherapy (IO) has established its role in the treatment algorithm of most cancer subtypes as a powerful therapeutic modality. Laboratory cancer research has focused on anti-tumor immunity and different ways that could eventually manipulate the immune system in favor of cancer patients. Given the rapid incorporation of new IO drugs in the already established practice, potential synergistic combinations between RT and IO are being explored. The immuno-modulatory (immunogenic cell death and radio-vaccination) effects of RT have been under investigation since the first decades of the 20th century [2]. Understanding these interactions between RT and the immune system would be a significant step towards the more effective utilization of both treatment modalities.

Rectal carcinomas comprise about one-third of colorectal cancer malignancies reported annually, with the estimate of new rectal cancer cases reaching up to 46,000 for the year 2023 in the US [5]. The gold-standard treatment for locoregionally advanced rectal cancer is neoadjuvant short-course RT or long-course chemoradiotherapy (CRT) followed by surgery and adjuvant chemotherapy. Total neoadjuvant therapy (TNT), the completion of RT and all courses of chemotherapy before surgery, has also been established as an equally effective approach that has been gaining more and more ground in recent years. Although IO’s exact role in rectal cancer treatment has not been determined to date, the 100% complete clinical response rates achieved with anti-PD1 treatment (dostarlimab) in 12 stage II–III rectal cancer patients with mismatch-repair (MMR)-deficient tumors have suggested that the exploitation of anti-tumor immunity could significantly improve rectal cancer patient prognosis [6]. 

In this review, we present and discuss studies conducted on immune response in patients with rectal cancer, assessing its impact on survival. Moreover, we focus on research dealing with the complex interactions between neoadjuvant RT and CRT with the host immune response and the outcome of therapy, showing the potential role of IO in the treatment of this cancer subtype. Since a number of published studies do not discriminate between rectal and colon cancer for several important biomarkers, but rather refer to colorectal carcinoma, selected interesting reports are included in the current review to provide an eventual trend in the absence of focused studies on rectal cancer. It is stressed that colon carcinomas have a distinct etiology and molecular carcinogenesis are linked to better overall outcome and are subject to an entirely different treatment approach, which is mainly based on postoperative chemotherapy without radiotherapy [7,8]. 

## 2. Tumor-Infiltrating Lymphocytes (TILs)

### 2.1. Overall TIL-Density

Rectal carcinomas, like any human tumor, are readily recognized by peripheral lymphocytes and monocytes that infiltrate the tumor stroma. Chemokine secretion by cancer and stroma cells greatly contributes to the attraction of inflammatory cells from the bloodstream [9]. One of the first studies focusing on the appraisal of TIL-density in hematoxylin and eosin tissue sections from 98 patients with rectal cancer found a striking difference among cases, with a TIL-density ranging from less than 10 to more than 100 lymphocytes per ×40 high-power optical field [10]. Low TIL density was linked with advanced T- and N-stage, as well as distant metastasis. Patients with high TIL density had a two-fold better 14-year survival. Subsequent studies also confirmed the ominous prognostic role of the poor lymphocytic infiltration of colorectal carcinomas [11,12,13,14,15,16]. All these studies included a significant proportion of rectal and colon carcinomas, but there has been no separate analysis. Moreover, there has been no specific comment on the treatment applied to patients. Nevertheless, it is assumed that low TIL-density is linked to advanced stage and poor postoperative prognosis in both colon and rectal adenocarcinomas. 

### 2.2. Overall T-Cell Density

Using broad T-cell-recognizing markers, such as CD3 and CD45RO, recent studies have demonstrated longer disease-free survival (DFS) and overall survival (OS) among patients with rectal carcinomas densely infiltrated with T-cells. A study by McMullen et al. focused, among other markers, on CD3+ T-cells accumulating on tumor-associated lymphoid nodules, showing that dense CD3+ TIL-density is linked with improved survival independently of disease stage [17]. In a study by Richards et al. that included 129 rectal carcinomas, CD3+ TIL-density, whether in the invasive margin, inner stroma or cancer cell nests, was correlated with significantly better prognosis [18]. Dahlin et al., in a series of colorectal carcinomas that included 110 rectal carcinomas treated with preoperative short-course RT, displayed that high CD3+ TIL-density in their stroma was linked with a two-fold better 5-year cancer-specific survival [19]. Another study on 185 patients with locally advanced rectal cancer treated with 10 fractions of 3 Gy before total mesorectal excision (TME) examined the CD45RO+ TIL-density in tumor tissues [20]. High TIL-density was associated with increased T- and N-downstaging after RT, and with better prognosis in multivariate analysis. 

A list of lymphocyte and monocyte subtypes identified with specific markers commonly used in translational studies is provided in Appendix A.

### 2.3. T-Cell Subtype Density

Further studies focused on the infiltration by T-cell subtypes, using immunohistochemistry to detect CD4+ memory/regulatory, CD8+ cytotoxic, or FOXP3+ regulatory T-cells. In 1998, Matsuda et al. observed a higher infiltration of CD4+ and TCRαβ+ cells in malignant colorectal tissues compared to predominant CD8+ and TCRγδ+ cell populations in normal epithelium, suggesting a significant entanglement of the immune system [21].

#### 2.3.1. Effects of RT

RT strongly affects the density and subtypes of lymphocytes that infiltrate tumors. Short-course RT and long-course CRT have been shown to increase the number of T-lymphocytes in the tumor microenvironment of locally advanced rectal carcinomas, although no significant alterations were noted in the CD4+ and CD8+ lymphocyte counts [22]. Shinto et al. prospectively analyzed 93 patients with rectal cancer who were treated with neoadjuvant RT (20 Gy in four fractions), together with oral tegafur/uracil, followed by surgery [23]. The immunostaining of pre-CRT and postoperative tumor specimens was demonstrated to lead to a significant increase in CD8+ TILs after CRT, and 5-year recurrence-free survival rates were improved in this patient subpopulation (87.5% vs. 57.8%). No changes were observed in the number of FOXP3+ TILs. Similar analyses of pre-CRT and postoperative surgical rectal cancer specimens also reported on the augmentation of CD8+ T-cell numbers after CRT [24,25,26,27]. In addition, a comparable effect was noted in CD4+ T-cell populations, while FOXP3+ TIL counts were stable [25]. Contrary to the aforementioned results, Posselt et al. showed a significant reduction in FOXP3+ expression levels in postoperative rectal cancer specimens of patients previously treated with CRT. CD8+ cells were not affected [28]. An analogous impact of CRT to FOXP3+ cell counts was documented by Rudolf et al., but CD8+ cell numbers also declined [29]. An increase in the CD8+/Granzyme B+ cytotoxic T cell ratio after CRT could eventually have a beneficial effect on prognosis [30]. 

The available studies comparing the differential effect of short-course RT or long-course CRT on the immune profile of rectal carcinomas are limited. In 2011, Dahlin et al. displayed a smaller probability of a high CD3+ score in surgical rectal cancer specimens post short-course RT when compared to the long-course RT schedule (25 fractions-2Gy/f) [19]. In the LYMPHOREC study, neoadjuvant short-course RT was associated with a lower CD8+/FOXP3+ TIL ratio, which was eventually defined as longer OS and progression-free survival (PFS) [31].

#### 2.3.2. Prognostic Relevance

Additional reports have been published on the role that T-lymphocytes subtypes that infiltrate rectal carcinomas play in treatment outcome. High numbers of CD8+, presumed to be cytotoxic T-cells, in rectal cancer tissues have been associated with low metastatic potential [32]. Anitei et al. examined the CD8+ TIL-density in postoperative tissue specimens from patients treated with neoadjuvant CRT who had residual disease and in pre-CRT biopsies [33]. High CD3+ and CD8+ TIL-densities in pre-RT biopsies were linked with increased downstaging, and also with a three-fold higher DFS. A study by Berntsson et al. examined CD3+, CD8+ and FOXP3+ TIL density in a series of colorectal carcinomas that included 209 patients with rectal cancer [34]. CD3+ TIL-density was associated with better prognosis, but CD8+ and FOPX3+ TIL-densities had prognostic relevance only in colon cancer cases. Schollbach et al. reported on 106 rectal cancer patients treated with neoadjuvant CRT and displayed that high CD8+ TIL-density in surgical specimens was linked with lower yp-T-stage and better prognosis [35]. 

Patients with increased CD4+ and CD8+ T-cells also showed a trend towards improved outcome in a study by Imaizumi et al. [36]. Moreover, a statistically significant correlation between better response to neoadjuvant therapy and increased CD4+ and CD8+ T-cell infiltration in pretreatment rectal cancer biopsies was demonstrated [37,38]. A higher density of FOXP3+ T cells could also be predictive of PFS and OS [28,31,39]. On the other hand, Mcoy et al. [40] found no association between any T-cell subpopulation (CD3+, CD8+ or FOXP3+) and response to neoadjuvant treatment, while FOXP3+ T-cells appeared to be inversely related to therapeutic outcome in a different study [41]. Similarly, rectal cancer patients with a lower infiltration of FOXP3+ T-cells in pretreatment biopsies responded better to neoadjuvant chemoradiation and had better prognosis [42]. 

Table 1 and Table 2 summarize the translational studies examining the role of lymphocytes in rectal cancer response to chemoradiotherapy and patient prognosis, as well as changes in immune response and immune checkpoint molecules’ expression in rectal cancer after radiotherapy/chemoradiotherapy, respectively.

### 2.4. Mismatch Repair Deficiency, Microsatellite Instability and TILs

The mismatch repair (MMR) system of nuclear enzymes is responsible for repairing the DNA damage induced by exogenous chemical and physical damage [43]. The repair of base–base mismatches or deletions and nucleotide insertion sites starts with the binding of the MutS heterodimer (composed of the MSH2 and MSH6 proteins) to the damaged DNA areas, followed by interaction with the MutL heterodimer (composed of the MLH1 and PMS2 proteins). Epigenetic silencing of such genes leads to loss of cell cycle arrest and apoptosis pathways, resulting in a continuous accumulation of gene mutations in the genome of cancer cells and the expression of high levels of mutant proteins. Consequently, foreign peptides are highly expressed and recognized by dendritic cells and educated cytotoxic T-cells. Such high-mutation-loaded tumors are vulnerable to anti-PD-L1/PD-1 immunotherapy, as shown by a clinical trial where 100% of MMR-deficient rectal carcinomas responded completely to dostarlimab [6].

The role of MMR deficiency in the response of rectal tumors to CRT is unclear. A study by de Rosa et al. showed the high effectiveness of neoadjuvant CRT in a series of 62 rectal cancer patients [44]. Other studies, however, displayed no association with CRT efficacy [45], or even resistance to CRT [46]. It is anticipated that forthcoming clinical trials will reveal the role of MMR status when tested together with TIL-density as an important tool to guide immuno-radiotherapy combinations for rectal cancer.

Microsatellite instability (MSI), the repeated sequence of 1–6 nucleotides, often eventually characterizes rectal tumors [47] as a result of DNA slippage during the process of replication [48]. Ninety-five percent of carcinomas with MMR deficiency also have MSI [49]. Colorectal tumors with MSI more frequently demonstrate high levels of neo-antigens and are immunologically hot tumors [50]. Nosho et al. found a direct association between MSI and high CD45RO+ TIL-density, with the latter being linked with better prognosis [51]. Nevertheless, even patients with tumors bearing microsatellite stability have a better prognosis when highly infiltrated by T cells or CD8+ TILs [19,52].

## 3. Immune Checkpoint and Immune-Related Molecule Expression

### 3.1. PD-1/PD-L1 Pathway

The PD-1/PD-L1 is one of the best-studied immune checkpoint pathways involved in cancer escape from immune surveillance. Programmed cell death 1 (PD-1; CD279) is a surface protein of T- and B- cells participating in the prevention of an over-reactive immune response that would damage normal tissues and lead to autoimmune diseases. Its ligand, PD-L1, expressed by regulatory immune cells and also cancer cells, binds to PD-1 on cytotoxic T-cells and blocks their activity [53]. In the last decade, numerous monoclonal antibodies targeting the PD-1 and its ligand PD-L1 have been approved for the treatment of all types of human cancer. In colorectal cancer, such antibodies have been approved for the treatment of cases with MMR deficiency and microsatellite instability [54]. The striking clinical evidence of dostarlimab anti-PD1 monotherapy eliminating 100% of locally advanced rectal cancer in a small cohort of patients with MMR-deficient rectal tumors may revolutionize our therapeutic policies against rectal cancer [6].

#### 3.1.1. Effects of RT

RT seems to directly affect the expression of PD-L1 by cancer cells and infiltrating immune cells (Table 2). Studies on rectal cancer patients demonstrated that PD-L1 cancer cell expression was augmented after neoadjuvant CRT [27,55,56]. For example, Chen et al. noted a significant increase in cancer cell PD-L1 expression after CRT (50% vs. 63% before and after CRT, respectively) [27]. Lim et al. also investigated PD-L1 cancer cell expression and CD8+ TILs status on pre- and post-CRT rectal cancer tissues of 123 patients and displayed a robust increase in both parameters [57]. Moreover, PD-L1-expressing TILs were shown to be affected by CRT, as an increase in their percentages was observed post treatment (from 31.7% to 49.2%). A positive correlation between the presence of PD-L1+ TILs and a high CD8+ TIL density was noted [58]. In contrast to the above studies, Huemer et al. found that PD-L1 is significantly down-regulated in post-RT cancer tissues, both as assessed in cancer cells (tumor proportion score (TPS) and as a combined score with the stroma (combined positive score (CPS)) [59]. As far as RT fractionation is concerned, there was no distinctive effect of short- or long-course RT on PD-L1+ cells [60]. Finally, Tominaga et al. noted increased soluble PD-L1 and stable PD-1 levels in the serum of rectal cancer patients treated with neoadjuvant CRT [61]. 

#### 3.1.2. Prognostic Relevance

As PD-L1 expression by cancer cells and regulatory immune cells is expected to repress anti-tumor immune responses, translational studies have searched for the role of PD-1/PD-L1 pathway in the prognosis of rectal cancer patients (Table 3). Chen et al. performed an immunostaining of PD-L1 in the pretreatment biopsies and post-CRT surgical material of 112 rectal cancer patients [27]. Both DFS and OS were significantly longer in patients with high tumor PD-L1, either before or after CRT. A similar finding linking high PD-L1 expression with a survival benefit was reported by Boustani et al. and Chiang et al. [56,60]. In addition, Huemer et al. noted that pre-RT CPS was linked with a better response to CRT, which was not verified for TPS [59]. Nevertheless, a TPS of higher than 1% was also associated with better survival.

This positive association between PD-L1 expression and prognosis, however, is not a consistent observation in the literature. A retrospective study on 68 patients with rectal cancer treated with preoperative RT assessed tumor PD-L1 expression and its effect on survival [62]. Although the number of rectal specimens with PD-L1 expression was low, there was a clear correlation between PD-L1+ tumor cells after RT and poor local recurrence-free survival, which was also confirmed in multivariate analysis. Saigusa et al. confirmed this detrimental effect of high tumor PD-L1 expression after CRT on recurrence-free survival and OS [63]. Of interest, PD-L1-positive tumors were significantly linked with vascular invasion. Lim et al. also found that a sustained high PD-L1 expression during CRT was associated with worse prognosis [57]. Supportive of the role of PD-L1 in defining tumor aggressiveness, a study by Miller et al. suggested that PD-L1 may be responsible for the progression of dysplastic rectal adenomas to carcinomas and their acquired invasive properties through the evasion of immunosurveillance [64]. Other studies in colorectal cancer also support poor prognosis in patients with high PD-L1 cancer cell expression [65,66].

PD-L1-expressing lymphocytes may have an independent role in the response of rectal cancer to CRT and the prognosis of patients. Park et al. reported that the density of tumor PD-L1+ lymphocytes, alongside the overall TIL-density and low CD4+ TIL-density in pretreatment biopsies, could determine complete tumor response to RT [67]. Moreover, TILs expressing PD-1 have been shown to be predictive of better OS and DFS in rectal cancer patients [68]. In a published study that does not discriminate between rectal and colon tumor anatomical sites, high PD-1+ TIL-density has been correlated with better prognosis [69]. Similar observations have been reported for high PD-L1+ TIL-density [70,71].

It is important to underline that the sole assessment of immune checkpoint molecules expression on tissue samples, regardless of the type of cells expressing them, could lead to conflicting results. *PD-L1* is also an inducible gene, regulated by the microenvironmental conditions (e.g., hypoxia) or cytokines released by inflammatory and stroma cells [72,73]. Thus, TILs expressing PD-L1 could be evidence of a high lymphocytic infiltration in the tumor microenvironment and subsequently better prognosis, while PD-L1+ tumor cells attempt to evade immunosurveillance, invade and promote metastasis. Rather than focusing on surgical rectal specimens, Tominaga et al. measured serum-soluble PD-L1 and PD-1 levels before and after CRT in patients with locally advanced rectal cancer. While serum PD-1 levels were stable after treatment, PD-L1 levels were significantly higher and a trend towards worse DFS was noted in these patients [61]. Systemic immune response and modulation after CRT may play an important role in the intratumoral immune response. For example, FOLFOX chemotherapy enhances CD8-mediated cytotoxic response in colorectal tumors [74], and RT induced-radio-vaccination triggers a large cascade of immune responses that may overcome the adverse role of PD-L1 expression [2].

### 3.2. The CTLA-4/CD80-86/CD28 Pathway

Another important immune checkpoint pathway is related to the CTLA-4 molecule expressed on the T-cell membrane. The CD80 (B7-1) and CD86 (B7-2) checkpoint proteins expressed by immune and cancer cells bind to CTLA-4 on T-cells and repress TCR/HLA-class-I-mediated activation [75]. The blockage of CTLA-4 with specific monoclonal antibodies allows for the T-cell CD28 protein to bind to CD80-CD86, which activates T-cells, promoting cancer cell lysis. The ipilimumab anti-CTLA-4 monoclonal antibody has been widely applied in clinical practice for the treatment of melanoma and lung carcinoma [76].

Limited data are available regarding the role of the CTLA-4 immune checkpoint pathway in rectal cancer as far as prognosis and response to treatment are concerned (Table 3). Peyravian et al. reported high mRNA levels of CD86 in adenomatous polyps, and thus suggested the utilization of this biomarker as a means to distinguish them from hyperplastic polyps, hinting at the potential role of CD86 in malignant transformation [77]. High levels of CD80 also characterize pre-neoplastic colon lesions [78,79]. Moreover, the presence of a specific CD86 gene polymorphism has been linked with an increased risk of colorectal cancer and worse OS for colon patients, but not for rectal cancer patients [80]. Kitsou et al. analyzed next-generation sequencing (NGS) data of 453 patients with colon and rectal cancer [81]. A trend towards longer OS was noted for patients with high CTLA-4 and PD-1 expression levels in T-cells. A more recent study assessing CTLA-4 and CD86 expression in TILs in 255 rectal carcinomas previously treated with CRT has provided insights into the role of this immune checkpoint pathway [82]. CTLA-4 and CD86 expression was observed in 69% and 14.1% of cases, respectively. Higher CTLA-4+ lymphocyte density was associated with longer PFS and metastasis-free survival. In contrast, CD86 expression by TILs was an independent negative prognostic factor. Regarding CD80/86 expression by rectal cancer cells and its role in response to CRT and prognosis, there are no available translational studies.

### 3.3. The CD47-SIRPα ‘Don’t Eat Me’ Pathway

Macrophage-mediated cancer cell phagocytosis is important for the clearance of irradiated tumors [83,84]. The dense infiltration of the rectal cancer stroma with CD68+ macrophages has been linked with a favorable prognosis in colorectal cancer [85,86]. Nevertheless, a study by Kitagawa et al. on pre-treatment biopsies from 275 patients with rectal cancer treated with neoadjuvant CRT showed that low CD68+ macrophage counts were more frequently recorded in tumors that poorly responded to CRT [87]. Similar findings were reported by Liu et al. in a series of 191 rectal cancer patients treated with preoperative CRT [88] (Table 1). As tumor-associated macrophages (TAMs) can be polarized towards M1-effector or M2-immunosuppressive phenotype, their role in response to RT and prognosis may be differentially driven by the TAM-subtype prevalence [89]. An assessment of M1 vs. M2 macrophage density in the tumor environment could, therefore, better predict rectal cancer responsiveness to RT and treatment outcome.

The phagocytic activity of macrophages is regulated by the CD47/SIRPα axis. Normal cells express the CD47 protein that blocks phagocytosis by binding to the signal regulatory protein alpha (SIRPα) membrane protein on macrophages. Cancer cells may also overexpress CD47, providing a ‘don’t eat me’ signal to escape macrophage attention [90]. Therapeutic monocolonal antibodies targeting this pathway are under development [91,92]. In experimental studies in colorectal cancer, CD47 is up-regulated after irradiation in surviving cancer cells, and this has been confirmed in clinical studies, where CD47 is significantly increased in surgical specimens from patients with rectal cancer treated with short-course RT [93]. The blockade of CD47 with anti-CD47 or anti-SIRPα antibodies resulted in increased rates of experimental tumor eradication after RT. Abscopal effects were also detected; after bilateral tumor implantation and the irradiation of only one lesion, high rates of eradication of both tumors were noted. Combination of RT with anti-SIRPα therapy enhanced the phagocytosis of irradiated cancer cells. 

Although there are no data regarding the predictive and prognostic role of CD47 in rectal cancer patients treated with CRT, two large studies in colorectal cancer confirm an ominous role of CD47. In a study by Sugimura-Nagata et al., a series of 269 colorectal carcinomas were examined, reporting CD47 expression in 35% of cases. This group of patients had worse prognosis [94]. A similar study in 328 colorectal adenocarcinomas treated with surgery demonstrated the overexpression of CD47 in 16% of patients, which was associated with node involvement, tumor budding and poor recurrence-free survival [95].

The above data strongly support the idea that the CD47/SIRPα axis plays an important role in rectal cancer resistance to CRT, and that post-irradiation tumor clearance through macrophage phagocytosis can be enhanced by the therapeutic targeting of this pathway.

### 3.4. HLA-Class-I Molecules

T-cell mediated anti-tumor immunity is significantly driven by the recognition of cancer antigens presented on the cell surface via HLA-class-I molecules by dendritic and cytotoxic T-cells. Loss of HLA-class-I molecules expression, which can be attributed to epigenetic pathways and the mutations of genes involved in the HLA-class-I complex formation, is a process that malignant cells utilize to evade immune-surveillance [96]. Experimental and clinical studies suggest that cancer cell irradiation upregulates HLA-class-I molecules, which may prove important in immuno-RT combinations [2,97,98]. Furthermore, cancer cells suppress HLA-class-I expression during IO, leading to immuno-resistance, while their re-expression after RT can restore IO efficacy in experimental models [99].

Rectal carcinomas have been shown to exhibit a loss or down-regulation of HLA-class-I molecules in more than a third of cases, which is eventually related to loss of differentiation [100,101]. In a study by Sato et al., the combination of neoadjuvant CRT with hyperthermia led to upregulation of HLA-class-I molecules post treatment, suggesting a favorable effect of treatment as a result of cancer cells’ exposure to immune recognition [102]. No association between HLA-class-I expression and prognosis was found in multivariate analysis (Table 2).

A large study on 1135 rectal carcinomas reported that low HLA-class-I expression was associated with worse OS and DFS, whether or not patients had received RT [103]. Worse prognosis in an analysis of 495 rectal cancer patients, whose tumors exhibited suppressed HLA-class I expression, was reported by Reimers et al. [39]. More recently, Michelakos et al. reported a pooled analysis from 5 studies on 1243 patients with rectal cancer, suggesting that high HLA-B/C expression was an independent factor of better prognosis [104] (Table 3).

**Table 1:** Translational studies examining the role of lymphocytes and macrophages in rectal cancer response to chemoradiotherapy and patient prognosis (studies that did not discriminate between colon and rectal cancer are not reported).

Author (Year), Reference	No. of Cases	Biomarker	Main Findings
**Lymphocytes**			
Ropponen et al. (1997) [10]	98	TIL-density	High TIL-density was linked with earlier T,N stage and better survival
McMullen et al. (2010) [17]	40	CD3+ T-cell density	High CD3+ T-cell density was linked with improved survival, regardless of disease stage
Richards et al. (2014) [18]	129	CD3+ T-cell density	High CD3+ T-cell density was associated with better prognosis
Dahlin et al. (2011) [19]	110	CD3+ T-cell density	High CD3+ T-cell density defined longer survival
Wang et al. (2015) [20]	185	CD45RO+ T-cell density	High post-CRT CD45RO+ T-cell density was linked with improved downstaging and better prognosis
Shinto et al. (2014) [23]	93	CD8+ T-cell density	Increased CD8+ T-cell counts after CRT related with better prognosis
Anitei et al. (2014) [33]	111	CD3+ and CD8+ T-cell density	High pre-RT CD3+ and CD8+ T-cell density was correlated with improved downstaging after RT and better DFS
Berntsson et al. (2017) [34]	209	CD3+, CD8+ and FOXP3+ T-cell density	High CD3+ T-cell density was correlated with better prognosis
Schollbach et al. (2019) [35]	106	CD8+ T-cell density	High post-CRT CD8+ T-cell density was associated with better downstaging and prognosis
Imaizumi et al. (2020) [36]	188	CD4+ and CD8+ T-cell density	High post-CRT CD4+ and CD8+ T-cell density was linked with better prognosis
Yasuda et al. (2011) [37]	48	CD4+ and CD8+ T-cell density	High pre-CRT CD4+ and CD8+ T-cell density was linked with improved downstaging and prognosis
Lai et al. (2020) [38]	134	CD4+ and CD8+ T-cell density	High pre-CRT CD4+ and CD8+ T-cell density was linked with improved downstaging
Posselt et al. (2016) [28]	202	CD8+ and FOXP3+ T-cell density	High post-CRT FOXP3+ T-cell density was linked with better prognosis
Mirjolet et al. (2017) [31]	237	CD8+ to FOXP3+ T-cell ratio	Low post-RT CD8+ to FOXP3+ T-cell density was linked with better prognosis
Reimers et al. (2014) [39]	495	FOXP3+ T-cell density	High FOXP3+ T-cell density was associated with better prognosis
Mcoy et al. (2017) [40]	106	CD3+, CD8+ and FOXP3+ T-cell density	No association of any T-cell density assessed before CRT with clinical outcome
Zhang et al. (2019) [41]	109	CD4+, CD8+ and FOXP3+ T-cell density	High CD4+ and CD8+ and low FOXP3+ T-cell density correlated with better treatment response and prognosis
Zaghloul et al. (2021) [42]	50	CD8+ and FOXP3+ T-cell density	High pre-CRT CD8+ and low FOXP3+ T-cell density were associated with improved downstaging
**Macrophages**			
Kitagawa et al. (2022) [87]	275	CD68+ macrophage density	High pre-CRT CD68+ macrophage density was linked with better response to CRT
Liu et al. (2021) [88]	191	CD68+ macrophage density	Low pre-CRT CD68+ macrophage density was correlated with poor response. Low pre- to post-CRT CD68+ macrophage ratio was linked with improved prognosis

Abbreviations: TIL = tumor-infiltrating lymphocytes, RT = radiotherapy, CRT = chemoradiotherapy.

**Table 2:** Translational studies examining changes in immune response and immune checkpoint molecule expression in rectal cancer after radiotherapy/chemoradiotherapy.

Author (Year), Reference	No. of Cases	Biomarker	Main Findings
**Lymphocytes**			
Lim et al. (2014) [22]	52	T-cell counts, CD4, CD8	Increased T-cell infiltration after RT/CRT. No change in CD4+/CD8+ relative lymphocyte counts
Shinto et al. (2014) [23]	93	CD8+ and FOXP3+ T-cell density	Increased CD8+ T-cell density after CRT. FOXP3+ T-cell density remained unaltered
Teng et al. (2015) [24]	136	CD8+ T-cell density	Increased CD8+ T-cell density after CRT
Teng et al. (2015) [25]	62	CD8+, CD4+ and FOXP3+ T-cell density	Increased CD8+ and CD4+ T-cell density after CRT. FOXP3+ T-cell density remained unaltered
Matsutani et al. (2018) [26]	64	CD8+ T-cell density	Increased CD8+ T-cell density after CRT
Chen et al. (2019) [27]	112	CD8+ T-cell density	Increased CD8+ T-cell density after CRT
Posselt et al. (2016) [28]	202	CD8+ and FOXP3+ T-cell density	Reduction in FOXP3+ T-cell density after CRT. CD8+ T-cell density remained unaltered
Rudolf et al. (2016) [29]	191	CD8+ and FOXP3+ T-cell density	Reduction in CD8+ and FOXP3+ T-cell density after CRT.
Jarosch et al. (2017) [30]	130	CD8+/GrzB+ to CD8+ T-cell ratio	Increased ratio after CRT.
Mirjolet et al. (2017) [31]	237	CD8+ to FOXP3+ T-cell ratio	Decreased ratio after RT.
Lim et al. (2017) [57]	123	CD8+ T-cell density	Increased CD8+ T-cell density after CRT
**PD-1/PD-L1 pathway**			
Chen et al. (2019) [27]	112	PD-L1 cancer cell expression	Increased PD-L1 cancer cell expression after CRT
Hech et al. (2016) [55]	103	PD-L1 cancer cell expression	Increased PD-L1 cancer cell expression after CRT
Chiang et al. (2019) [56]	104	PD-L1 cancer cell expression	Increased PD-L1 cancer cell expression after CRT
Lim et al. (2017) [57]	123	PD-L1 cancer cell expression	Increased PD-L1 cancer cell expression after CRT
Ogura et al. (2018) [58]	287	PD-L1+ T-cell density	Increased PD-L1+ T-cell density after CRT. Direct correlation with CD8+ T-cell density
Huemer et al. (2020) [59]	72	PD-L1 cancer cell (TPS) and inflammatory cell expression (CPS)	Decreased TPS and CPS after CRT
Tominaga et al. (2019) [61]	117	Serum soluble PD-1 and PD-L1 levels	Increased PD-L1 and stable PD-1 levels after CRT
Tayshetye (2022) [105]	41	PD-L1+ TIL-density	Stable PD-L1+ TIL density after CRT
**CTLA-4/CD80-86/CD28 pathway**			
No studies available			
**CD47/SIRPα pathway**			
No studies available			
**HLA-class-I expression**			
Sato et al. (2014) [102]	78	HLA-class-I cancer cell expression	Increased HLA-class-I cancer cell expression after hyperthermic CRT.
**Other immune-related pathways**			
Peng et al. (2021) [106]	76	LAG-3+ and TIM-3+ TIL-density	Increased LAG-3+ and decreased TIM-3+ TIL density after CRT
Tayshetye (2022) [105]	41	OX40+, TIM-3+ and LAG-3+ TIL-density	Increased OX40+ and LAG-3+ TIL density after CRT. TIM-3+ TIL density was unaltered

Abbreviations: TIL = tumor-infiltrating lymphocytes, RT = radiotherapy, CRT = chemoradiotherapy, TPS = tumor proportion score, CPS = combined positive score.

**Table 3:** Translational studies examining the role of immune- and metabolite-related molecule expression in rectal cancer response to chemoradiotherapy and patient prognosis (studies that did not discriminate between colon and rectal cancer are not reported).

Author (Year), Reference	No. of Cases	Biomarker	Main Findings
**PD-1/PD-L1 pathway**			
Chen et al. (2019) [27]	112	PD-L1 cancer cell expression	High pre- and post-CRT PD-L1 cancer cell expression was linked with better prognosis
Boustani et al. (2020) [50]	74	PD-L1 cancer cell expression	High pre-CRT PD-L1 cancer cell expression was linked with better prognosis. No association of PD-L1 expression with CD8+ T-cell density
Chiang et al. (2019) [56]	104	PD-L1 cancer cell expression	High post-CRT PD-L1 cancer cell expression was associated with better prognosis
Huemer et al. (2020) [59]	72	PD-L1 cancer cell (TPS) and inflammatory cell expression (CPS)	High CPS was linked with improved downstaging, while high pre- and post-CRT TPS was linked with better prognosis
Shao et al. (2017) [62]	68	PD-L1 cancer cell expression	High post-RT PD-L1 cancer cell expression was linked with poor prognosis
Saigusa et al. (2016) [63]	90	PD-L1 cancer cell expression	High post-CRT PD-L1 cancer cell expression was linked with poor prognosis. High PD-L1 expression was linked with low CD8+ T-cell density
Lim et al. (2017) [57]	123	PD-L1 cancer cell expression	Sustained high PD-L1 cancer cell expression after CRT was linked with worse prognosis
Park et al. (2017) [67]	75	PD-L1+ TIL-density	High pre-CRT PD-L1+ TIL density was linked with complete tumor regression
Gruber et al. (2020) [68]	75	PD-1+ and PD-L1+ TIL density	PD-1+ TIL density was linked with better prognosis
Tominaga et al. (2019) [61]	117	Serum soluble PD-1 and PD-L1 levels	High serum-soluble PD-L1 levels after CRT were with worse prognosis
Peng et al. (2021) [106]	76	PD-1+ TIL-density	High post-CRT PD-1+ TIL density was linked with better prognosis
**CTLA-4/CD28/CD80-86 pathway**			
Yin et al., (2022), [82]	255	CTLA-4 and CD86+ TIL-density	High post-CRT CTLA-4+ and low CD86+ TIL density were linked with better prognosis. High CTLA-4+ and CD8+ density were directly related to each other
**CD47/SIRPα pathway**			
No studies available			
**HLA-class-I expression**			
Sato et al. (2014) [102]	78	HLA-class-I cancer cell expression	No significant association with prognosis in multivariate analysis
Speetjens et al. (2008) [103]	1135	HLA-class-I cancer cell expression	Low HLA-class-I expression was linked with worse prognosis
Reimers et al. (2014) [39]	495	HLA-class-I cancer cell expression	Low HLA-class-I expression was linked with worse prognosis
Michelakos et al. (2022) [104]	1243	HLA-class-I cancer cell expression	Low HLA-B/C expression was linked with worse prognosis
**Other immune-related pathways**			
Peng et al. (2021) [106]	76	LAG-3+ and TIM-3+ TIL-density	High post-CRT LAG-3+ and TIM-3+ TIL density was linked with better prognosis
**Aminoacids and metabolites**			
Schollbach et al. (2019) [35]	106	IDO1 cancer cell and stroma expression	High IDO1 cancer cell and stroma expression was linked with better prognosis
Zhang et al. (2015) [107]	20	CD73+ cancer cell expression	High CD73 cancer cell expression was associated with poor prognosis
Zhang et al. (2015) [108]	17	CD39+ cancer cell expression	High CD39+ cancer cell expression was linked with better prognosis

Abbreviations: TIL = tumor-infiltrating lymphocytes, RT = radiotherapy, CRT = chemoradiotherapy, TPS = tumor proportion score, CPS = combined positive score.

### 3.5. Other Immune-Related Pathways

Other immune checkpoint pathways have attracted attention in the development of novel IO policies. LAG-3 (CD223) is an immunoglobulin expressed on exhausted T-cells and NK-cells, as well as dendritic cells [109]. The blockage of LAG-3 re-activates exhausted cytotoxic CD8+ T-cells and promotes the differentiation of CD4+ T-cells with helper activity [110,111]. The TIM-3 (CD366) is another immune checkpoint receptor expressed by both helper-CD4+ and cytotoxic-CD8+ T-cells [112]. TIM-3 activation blocks helper-response and the secretion of TNFα from T-cells and leads to T-cell exhaustion. Ligands such as Galectin-9, phosphatidylserine, HMGB 1 and CEACAM-1 can activate TIM-3. TIM-3 activation also favors M2-macrophage polarization and immunosuppressive functions. 

The role of LAG-3 and TIM-3 has not been extensively studied in rectal cancer (Table 2 and Table 3). Peng et al. investigated the expression of LAG-3 and TIM-3 in 76 rectal cancer patients treated with preoperative RT [106]. LAG-3-expressing immune cells increased in cancer tissues after RT, while TIM-3 decreased. The high LAG-3 and TIM-3 expression in immune cells was linked with better prognosis. In contrast to this study, other reports found clinical aggressive behavior and significantly poorer prognosis in colorectal cancer patients expressing LAG-3 and TIM-3 [113,114,115,116].

T-cell immunoreceptor with immunoglobulin and ITIM domain (TIGIT) plays a critical role in repressing adaptive and innate immune response [117]. TIGIT is activated by co-stimulatory receptors such as CD226/DNAM-1 and ligands (CD155, CD112), or can be inhibited by inhibitory receptors such as CD96 and CD112R. The inhibition of TIGIT enhances cytotoxic T-cell responses [118]. Studies on TIGIT expression in rectal cancer are not available. Nevertheless, a negative impact of TIGIT-expressing T-cells on the prognosis of patients with colorectal cancer has been suggested [119,120].

Activated CD4+ (memory and regulatory), cytotoxic-CD8+ T-cells and antigen-presenting cells also express two important immune checkpoint molecules: OX40 (CD134) and its ligand member of the TNF family OX40L (CD134L or CD252) [121]. OX40 is an IL-2-inducible protein. The OX40/OX40L axis amplifies antigen-specific T-cell proliferation, CD4+ memory T-cell activity, the adhesion and migration of T-cells, and the production of effector cytokines. Of interest, the OX40-mediated activation of T-cells renders them resistant to regulatory T-cells [122]. CRT induces OX40 and LAG-3 expression on lymphocytes infiltrating the irradiated rectal carcinomas [105], suggesting the activation of anti-tumor immune response. A large study on 441 colorectal cancer tumors analyzed on tissue microarrays suggested that tumor infiltration by OX40+ immune cells conferred a longer OS [123]. This observation has also been confirmed in other studies [124,125]. Novel IO agents targeting the OX40/OX40L axis are under investigation [126]. Such agents may play an important role as an adjunct therapy to RT for rectal cancer patients.

## 4. Aminoacids and Metabolites

The metabolism of certain amino acids has been implicated in immune suppression pathways in the tumor microenvironment. Indoleamine-2,3-dioxygenase (IDO1) and tryptophan-2,3-dioxygenase (TDO2) are involved in the metabolism of the essential aminoacid tryptophan to kynurenine. Kynurenine has potent immunosuppressive activities by inhibiting T-cell and NK-cell function and activating regulatory T-cells. The IDO1/kynurenine pathway has become a target for the development of IO molecules [127]. Crotti et al. reported high kynurenine levels in the plasma of rectal cancer patients [128]. Several studies in colorectal cancer have revealed contradictory results regarding the prognostic role of IDO1 expression [129,130,131]. In a report by Schollbach et al. [35], strong cancer cell and stroma IDO1 expression was confirmed in 38.5% of rectal tumors, and was related with intense CD8+ TIL-density and better outcome. In contrast, in a study on anal cancer patients treated with CRT, IDO1 expression defined a significantly worse prognosis [132].

The depletion of arginine, a semi-essential aminoacid, through arginase enzyme activity also has potent immunosuppressive effects, as arginine is crucial for T-cell proliferation and function [133]. Although studies on the role of arginine and arginine-related enzymes in rectal cancer are not available in the literature, the overexpression of arginase-1 defines poor prognosis in colorectal cancer [134]. Moreover, increasing arginase serum activity after surgery has been linked with the recurrence of colorectal carcinomas [135].

Adenosine is an immunosuppressive metabolite produced by the degradation of ATP released by cancer cells in the stroma. The enzymic activity of the ectonucleotidases CD73 and CD39 is responsible for ATP-to-adenosine conversion [136]. The blockage of CD73 in experimental mouse rectal cancer models has been shown to enhance RT efficacy and trigger abscopal effects [137]. CD73 is extensively expressed in human rectal cancer cells, and this is linked with poor prognosis, although CD73 expression in the tumor stroma predicts a better outcome [107]. CD73 expression in a large series of metastatic liver lesions from colorectal carcinomas was associated with poor response to chemotherapy and worse clinical outcome [138]. In addition, CD39 is strongly expressed by epithelial cells of rectal cancer and a link with better prognosis has been reported [108].

Further studies on amino acids and metabolites are expected to reveal important biomarkers of prognosis and response to therapy, and provide targets for the development of IO agents and combination policies for rectal cancer.

## 5. Systemic Immune Response

Although the expression of immune checkpoint molecules by cancer cells and their interaction with T-cell and monocyte subpopulations seem to affect the outcome of RT in rectal cancer, the systemic immune response is also a potent player in anti-tumor immunity. A study by Spitzer et al. suggested that the lack of a continuously active systemic immune response abrogates any intratumoral immune activity [139]. Shortly following IO, immune activation takes place within the tumor and peripherally (bone marrow, lymph nodes and blood). This systemic immunity feeds the intratumoral immune response and is essential to tumor eradication.

The lymphotoxic effects of RT may, therefore, counteract both its immune-modulatory effects and IO activity. Indeed, a decrease in lymphocyte counts is observed after the neoadjuvant treatment of rectal cancer patients. Tumor regression rates, DFS and OS were better when post-treatment total lymphocyte counts or lymphocytes to white blood cell ratios were higher [140,141,142,143]. The systemic immune-inflammation index (SII) (platelets x neutrophils/lymphocytes) has been utilized in recent years as a prognostic indicator for the most malignant tumors [144]. In this context, a low SII was shown to be associated with the higher complete response rates observed after neoadjuvant CRT in rectal cancer patients [145]. Unresponsiveness to neoadjuvant CRT has been displayed to be more common in patients with a high pretreatment-platelet-to-neutrophil ratio [146], while significantly better responses to CRT were observed in cases with low SII, neutrophil and platelet-to-lymphocyte ratios before therapy [147]. Finally, local relapse, DFS and OS were reported to be worse in rectal cancer patients exhibiting a high neutrophil-to-lymphocyte ratio as measured before or after neoadjuvant CRT [148,149].

The quality of circulating T-cells and monocytes has also been investigated in a limited number of studies on rectal cancer. Tojo et al. assessed the presence of PD-L1 expressing monocytes in the blood of rectal cancer patients [150]. A high percentage of this sub-population before and after CRT correlated with low T-cell counts and was significantly linked with poor response to therapy. PD-L1 expression, therefore, by regulatory immune cells in the systemic circulation, may disarm cytotoxic T-cells in the tumor periphery in parallel with a similar activity in the tumor microenvironment, and this occurs independently from cancer cell PD-L1-expression status. As mentioned earlier, an increase in serum PD-L1 levels has also been reported after CRT and has been associated with worse DFS in rectal cancer patients [61]. Thus, circulating immune checkpoint ligands may have a role in systemic immune response and the outcome of RT for rectal cancer patients.

A higher CD8+ T-cell content would be expected to favorably affect the efficacy of IO and RT. Indeed, a low CD4+/CD8+ T-cell ratio and CD4+ T-cell presence in the blood was linked with a favorable anti-PD-1 IO outcome for MMR-deficient colorectal carcinomas [151]. In addition, Wang et al. observed that decreased CD4+/CD8+ ratio and regulatory T-cell counts defined better response to RT in 108 patients with rectal cancer (only abstract available) [152]. Another study by Napolitano et al. suggested that an abundance of FOXP3+ regulatory T-cells after CRT was linked with the poor response of rectal tumors [153]. In contrast, Zhu et al. found that high levels of CD4+ circulating T-cells were correlated with better tumor response to RT and better prognosis [154].

## 6. Patient Derived Tumor Organoids

Beyond the analysis of original tissue biopsies and surgical specimens before and after CRT to assess the immune markers of response to RT that may also guide immunotherapy combinations, a novel technology, namely the development of patient derived tumor organoids (PDTOs) [155], may prove important for the individualization of therapy for rectal cancer. PDTOs are 3D tissue structures composed of cancer cells isolated from individual patient tumors. Such organoids can also be transplanted to animals, providing patient-derived xenograft models (PDTX).

PDTOs for immuno-RT combinations, however, demand the creation of complex organoids that simulate real tissues, with the co-culture of fibroblasts, endothelial cells, and, of course, immune cells. Such complex organoids are at an early stage of development and require the maintenance of immune cells infiltrating the original tumor or the separate development of tumor and lymphoid organoids that are afterwards brought in contact for a combined culture. Experimental studies on complex organoid interactions with immunotherapy have been summarized in a review article by Grönholm et al. [156].

Although there are no reported clinical studies on the usage of PDTOs for immune response evaluation and rectal cancer CRT outcome, encouraging results have been reported in preliminary studies using simple PDTOs, showing that their ex vivo sensitivity to irinotecan and CRT can predict clinical tumor responsiveness [157,158,159]. 

## 7. Conclusions

RT and CRT have a direct effect on the cancer cell expression of immune checkpoint molecules, and on the quantity and quality of lymphocytes and monocytes invading the irradiated rectal cancer tissue. Enhanced overall lymphocytic presence, especially infiltration by cytotoxic CD8+, PD-1+, and OX40+ lymphocytes, and activated macrophages, are important in the eradication of irradiated cancer cells. The up-regulation of PD-L1 and CD47 in rectal cancer cells, however, may block this effect. PD-L1/PD-1- and CD47/SIRPα-targeting ΙO could eventually unleash the anti-tumor potential of activated lymphocytes and monocytes. Research on aminoacid metabolism and other metabolites that strongly affect anti-cancer immunity is expected to provide critical targets to consider for rectal cancer immuno-RT. Furthermore, the systemic immune status and response emerge as important factors affecting treatment outcome. In this context, the lymphotoxic effects of RT should be considered when developing immuno-RT protocols for patients with locally advanced rectal cancer. 

## Appendix A

Lymphocyte and monocyte expression markers and their role in anti-tumor immunity.

**Table:**

Lymphocyte/Monocyte Markers	Lymphocyte Subtype	Function	Reference
CD3	T-cell co-receptor expressed in all T-cells. Additionally xpressed in pre-T-cell stem cells	Involved in both CD4+ and CD8+ T-cell activation	[160]
CD45RO	Expressed in activated and memory T-cells	Involved in T-cell activation	[161]
CD4	Expressed in naïve T-cells, but also in dendritic cells and monocytes	CD4-expressing T-cells can be differentiated to helper memory and regulatory T-cells, while CD4 expression is sustained	[162]
CD8	Expressed in T-cells	CD8+ are critical T-cells of the adaptive immunity. These are mainly cytotoxic T-cells that target virally infected and cancer cells	[163]
FOXP3	CD4+/CD25+ T-cells that have been differentiated to express FOXP3	FOXP3+ T-cells are regulatory T-cels (Tregs) that sustain immune tollerance	[164]
Granzyme B	CD8+ cytotoxic T-cells may express Granzyme B	Cytotoxic T-cells and NK cells use granzyme B to kill virally infected and cancer cells	[165]
PD-L1	Expressed by macrophages and a subset of regulatory lymphocytes	PD-L1 suppresses the adaptive responses of the immune system	[166]
PD-1	Expressed by cytotoxic T-cells and activated NK and B-cells, monocytes and dendritic cells	Involved in the inhibition of both adaptive and innate immunity	[166]
CTLA-4	Expressed by cytotoxic T-cells and Tregs	Binding to CD80/86 suppresses immune response	[167]
CD28	Expressed by cytotoxic T-cells	Activates T-cell activity when bound to CD80/86. CTLA4 is a competitive inhibitor of CD28	[167]
SIRPα	Expressed by macrophages	Blocks phagocytic activity when bound to CD47 on target cells	[168]
CD68	Expressed by macrophages and mononuclear cells	Involved in innate immunity	[169]
LAG3/CD223	Ligand of MHC-class-II. Expressed by activated T-cells, NK and B-cells and Dendritic cells	Negative regulator of proliferation and activation of T-cells. Sustains tollerogenic state of CD8+ T-cells	[170]
TIM3	Expressed on activated CD4+ and CD8+ T-cells but also in dendritic cells and other immune cells	Mediate CD8 T-cell exhaustion	[171]
TIGIT	Expressed by CD8+ T-cells and NK-cells	Blockage of TIGIT promotes T-cell proliferation and cytotoxic T-cell activity. Binding to CD155 on dendritic cells promotes their tolerogenic status	[117]
OX40/CD134	Expressed by activated T-cells, NK-cells and neutrophils	Binds to OX40L ligand. Involved in the survival of T-cells, the development of memory T-cells and the activation of dendritic cells. Inhibits FOXP3 expression	[172]
